# Diet and environment: unexplored influences on sleep quality in postural orthostatic tachycardia syndrome (POTS)

**DOI:** 10.1097/MS9.0000000000005070

**Published:** 2026-04-30

**Authors:** Syeda Samia Fatima, Syeda Nashrah Ayaz, Ahmed Asad Raza, Abedin Samadi

**Affiliations:** a Department of Medicine, Jinnah Sindh Medical University, Karachi, Pakistan; b Department of Medicine, Kabul University of Medical Sciences, Abu Ali ibn Sina, Kabul, Afghanistan

**Keywords:** autonomic dysfunction, diet, environmental factors, postural orthostatic tachycardia syndrome, sleep disturbances

## Abstract

Postural orthostatic tachycardia syndrome (POTS) is a chronic autonomic disorder characterized by an excessive increase in heart rate upon standing, accompanied by symptoms such as dizziness, fatigue, palpitations, cognitive impairment, and sleep disturbances. Sleep dysfunction is increasingly recognized as a significant contributor to symptom burden and reduced quality of life among individuals with POTS. Emerging evidence suggests that patients with POTS may experience heightened sympathetic activity during sleep, leading to disrupted autonomic regulation and fragmented sleep patterns. While the pathophysiological mechanisms underlying sleep disturbances in POTS are multifactorial, modifiable lifestyle factors – including dietary habits and environmental exposures – may play an underexplored role. Dietary factors such as high-fat meals or specific dietary patterns may influence sleep architecture and nocturnal awakenings, whereas interventions such as gluten-free diets have shown potential benefits in alleviating certain gastrointestinal and vasomotor symptoms associated with POTS. Environmental determinants, including chronic noise exposure and light pollution, can further disrupt circadian rhythms by impairing melatonin secretion and altering sleep quality. These disruptions may exacerbate autonomic imbalance, perpetuating daytime symptoms such as tachycardia and fatigue. Despite plausible mechanistic links, targeted research examining the influence of diet and environmental factors on sleep quality in POTS remains limited. Addressing these modifiable factors may provide novel lifestyle-based strategies to complement pharmacological management and improve patient outcomes.

Postural orthostatic tachycardia syndrome (POTS) is a chronic autonomic disorder wherein an excessive increase in heart rate occurs when standing up^[^1^]^, often associated with other symptoms including light-headedness, weakness, fatigue, brain fog, blurred vision, chest pain, shortness of breath, and nausea. Patients can also have trouble concentrating, shakiness or fainting spells, coldness, pain, and numbness in the extremities. Heightened sympathetic activation during orthostasis further contributes to palpitations, tremors, and sleep-related complaints^[^2,3^]^.

Evidence suggests that during sleep, POTS patients can enter a hyperadrenergic state, disrupting normal autonomic regulation and perpetuating a vicious cycle of poor sleep and worsened daytime symptoms^[^4^]^. Recently, sleep disturbances among individuals with POTS have gained attention, however, limited discussion exists on the influence of modifiable lifestyle factors on this critical aspect of the condition.

Modifiable factors such as diet and environmental impact sleep quality and have been studied in the general population. However there is absence of substantial literature for POTS patients specifically. Various studies have demonstrated that consuming fatty foods may cause mid night awakenings^[^5^]^. Also, gluten-free diet is seen to reduce various gastrointestinal and vasomotor symptoms^[^6^]^.

Environmental determinants represent another overlooked dimension. Chronic noise exposure has also been known to affect the quality of sleep in people, and because it makes him more prone to developing hypertension and other cardiovascular diseases, having them will make POTS more dysregulated^[^7^]^. Also, light pollution can interfere circadian rhythm by decrease melatonin secretion and thereby affecting the quality and quantity of sleep^[^8^]^. Since these factor can cause sleep disruption, it may make POTS-associated symptoms and autonomic functioning worse. Mechanisms common to sleep regulation and POTS, such as autonomic nervous system control, baroreceptor sensitivity, and circadian rhythms of cortisol secretion have also been identified. These pathways are responsive to external cues, such as dietary constituents and environmental exposures. Dysregulation in any of these inputs can result in fragmented sleep, which can increase sympathetic nervous system activity, compounding POTS symptoms like tachycardia, fatigue, and exercise intolerance^[^4,9^]^.

Despite these plausible connections there exists a lack of targeted studies evaluating this critical link. The impact of dietary interventions and environmental modifications on sleep quality in POTS patients must be studied for personalized lifestyle management beneficial for POTS patients. Addressing these factors can offer therapeutic strategies for POTS patients beyond pharmacological approach.

To ensure methodological transparency and ethical compliance in manuscript preparation, we adhered to the recently developed TITAN (Transparency in the Reporting of Artificial Intelligence) 2025 guidelines, which outline standards for responsible AI use in biomedical writing and publishing^[^10^]^.

## Data Availability

No datasets were generated or analyzed during the current study.

## Research registration unique identifying number (UIN)

Not available.
